# Markers of Inflammation and Fibrosis in the Orbital Fat/Connective Tissue of Patients with Graves' Orbitopathy: Clinical Implications

**DOI:** 10.1155/2014/412158

**Published:** 2014-09-17

**Authors:** Przemyslaw Pawlowski, Joanna Reszec, Anja Eckstein, Kristian Johnson, Andrzej Grzybowski, Lech Chyczewski, Janusz Mysliwiec

**Affiliations:** ^1^Department of Medical Pathomorphology, Cathedral of Biostructure, Medical University of Białystok, 13 Waszyngtona Street, 15-269 Białystok, Poland; ^2^Department of Pediatric Ophthalmology with Strabismus Treatment Unit, Medical University of Białystok, 17 Waszyngtona Street, 15-274 Białystok, Poland; ^3^Department of Ophthalmology, University Hospital Essen and University of Duisburg-Essen, Hufeland Straße 55, 45-122 Essen, Germany; ^4^Bayer Healtcare, Kaiser-Wilhelm-Allee 10, Leverkusen, 51-373 Nordshein-Westfalen, Germany; ^5^Department of Ophthalmology, Poznań City Hospital, 3 Szwajcarska Street, 61-285 Poznań, Poland; ^6^Department of Ophthalmology, University of Warmia and Mazury, 30 Warszawska Street, 10-082 Olsztyn, Poland; ^7^Department of Nuclear Medicine, Medical University of Białystok, 24A Skłodowskiej-Curie Street, 15-276 Białystok, Poland

## Abstract

*Purpose.* To assess FGF-*β*, TGF-*β*, and COX2 expression and immunocompetent cells in the orbital tissue of patients with severe and mild Graves' orbitopathy.* Patients and Methods.* Orbital tissue was taken from 27 patients with GO: (1) severe GO (*n* = 18), the mean clinical activity score (CAS) being 8.5 (SD 2.5); and (2) mild GO (*n* = 9), the mean CAS being 2.2 (SD 0.8), and from 10 individuals undergoing blepharoplasty. The expression of CD4+, CD8+, CD20+, and CD68 and FGF-*β*, TGF-*β*, and COX2 in the orbital tissue was evaluated by immunohistochemical methods.* Results.* We demonstrated predominant CD4+ T cells in severe GO. CD68 expression was observed in the fibrous connective area of mild GO and was robust in severe GO, while the prominent TGF-*β* expression was seen in all GO. Increased FGF-*β* expression was observed in the fibroblasts and adipocytes of severe GO. No expression of COX2 was found in patients with GO.* Conclusions.* Macrophages and CD4 T lymphocytes are both engaged in the active/severe and long stage of inflammation in the orbital tissue. FGF-*β* and TGF-*β* expression may contribute to tissue remodeling, fibrosis, and perpetuation of inflammation in the orbital tissue of GO especially in severe GO.

## 1. Introduction

Graves' orbitopathy (GO) is a disfiguring and sometimes blinding disease, characterized by inflammation and swelling of orbital tissues, with fibrosis and adipogenesis being predominant features [1].

Clearly, the vulnerability to disease manifestation most likely reflects the highly specialized function of the orbital tissue, a unique fat depot that cushions the globe [2]. Regensburg et al. found that, in GO patients, 25% have orbital fat and muscle volumes within an age-specific reference range. An increase of the fat volume, characterized by proptosis, is seen in approximately 14% of GO patients [3]. However, only 3 to 5% of patients with GO have severe disease with intense pain, inflammation, and sight-threatening corneal ulceration or compressive optic neuropathy [4].

The sparse mononuclear cell infiltrates are seen within the fatty connective tissue and the muscle endomysium [5, 6]. In the early inflammatory phase the majority of cells are T lymphocytes CD4+ and CD8+ and B lymphocytes are only occasionally seen [7, 8]. Macrophages influx is increased in the early disease and less so in late disease [9]. The inflammatory cells, T and B lymphocytes, and macrophages as well as mast cells infiltrating the orbit interact with orbital fibroblasts and amplify inflammatory/autoimmune reaction [9]. Activated human T lymphocytes by expressing cyclooxygenase-2 and producing prostaglandins drive human orbital fibroblast differentiation to adipocytes [10]. Moreover, engagement of CD40 on orbital fibroblasts triggers hyaluronan synthesis and activation of inflammatory cyclooxygenases [11].

**Figure 1:**
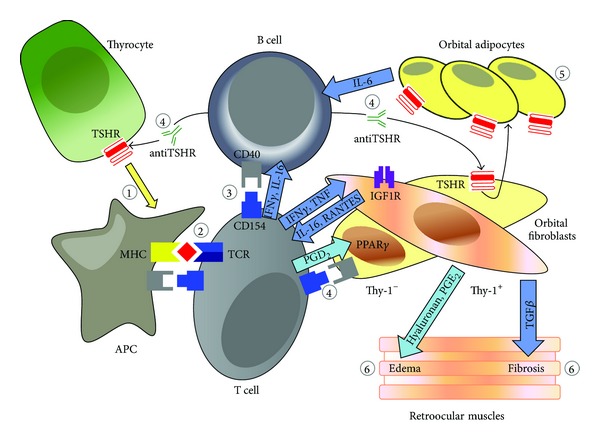
Immunopathogenesis of GO, highlighting the role of counterplay in the orbit between adipocytes, orbital fibroblast, and immunocompetent cells (lymphocytes T and B and macrophages) (adapted from a book chapter with the permission of Professor J. Mysliwiec).

When exposed to TGF-*β*, Thy-1^+^ fibroblast differentiates into myofibroblasts with prominent cytoplasmic actin filaments that can participate in inflammation, repair, and fibrosis and are responsible for tissue remodeling in GO [12, 13] (Figure 1).

TGF-*β* and other inflammatory mediators (IL-16, RANTES) elaborated by resident macrophages and fibroblasts trigger T cells migration or participate directly in local inflammation [14]. The inflammatory cyclooxygenase (COX2) is usually expressed at extremely low levels under normal basal physiology conditions. COX2 is expressed at higher levels in the orbital fibroadipose tissue in GO; there seems to be a positive correlation with increasing severity of ophthalmopathy, suggesting a possible relationship with COX2 expression and orbital inflammation in GO [15, 16].

Of the cell types residing in GO tissue preadipocytes and fibroblasts are most likely target and effectors cells of the orbital immune processes [12]. Fibrogenic growth factor, oxygen free radicals, and cytokines released from inflammatory cells act upon orbital preadipocytes, in para- or autocrine manner, stimulating adipogenesis, fibroblast proliferation, and glycosaminoglycan synthesis. Expression of FGF-*β* in the orbital fat/connective tissue with GO and a direct relation with higher CAS suggest its important role in severity of GO [17].

Therefore we decided to perform a study aimed at comparing leucocyte infiltration pattern and inflammatory (COX2) and profibrotic (FGF-*β*, TGF-*β*) cytokine milieu in the orbital fat/connective tissue of patients with mild and severe GO taken during decompression surgery.

## 2. Material and Methods

### 2.1. Patients and Controls

Human orbital tissue was obtained from 27 patients with GO (26 females and 1 male) classified according to the European Group on Graves' Orbitopathy, who underwent orbital decompression procedures, from the orbital tissue bank at the Department of Ophthalmology and University of Essen. The mean age of patients at the time of surgery was 44.5 years (range 26–47).

Control fat/connective tissues were derived from 10 individuals (9 females and 1 male) undergoing orbital surgery for blepharoplasty with no history of GO or any orbital inflammatory disease.

Surgical specimens of orbital fat/connective tissue were obtained and immediately snap-frozen in liquid nitrogen until use.

The clinical activity score of GO (CAS) was estimated according to Mourits et al. [18]. The severity of the eye disease was estimated using NOSPECS classification (no signs or no symptoms; only signs, no symptoms; signs only; proptosis; eye muscle involvement; corneal involvement; and sight visual acuity reduction) [19].

#### 2.1.1. Patients with Severe GO

Patients with severe GO (*n* = 18) (NOSPECS IV–VI) required orbital bony decompression due to optic nerve compression and limited extraocular muscle functions (22 specimens). Mean duration of thyroid disease was 2.5 (SD 1.5) years with 1.2 (SD 1.0) years for GO. Before surgery all patients had received >1 cycles of steroid regiment, and all but 2 received orbital irradiation. The mean clinical activity score was 8.5 (SD 2.5).

#### 2.1.2. Patients with Mild GO

Patients with mild GO (*n* = 9) (NOSPECS III-IV) underwent orbital bony decompression to reduce proptosis or for fat resection. Mean duration of thyroid disease was 3.9 (SD 2.0) years and 3.3 (SD −1.9) for GO. Steroid regiment and received orbital irradiation were similar for the patients with severe GO. The mean clinical activity score was 2.2 (SD 0.8).

### 2.2. Methods

Evaluation of proteins expression was done using immunohistochemical methods. Following the deparaffinisation and rehydration, epitope retrieval was carried out in the EnVision Flex Target Retrieval Solution (DAKO) in high pH. Endogenous peroxidases were blocked by incubating the sections in methanol and 3% hydrogen peroxidase for 20 minutes. Next slides were incubated with special types of antibodies (Table 1). Visualization reagent EnVision Flex (DAKO) was applied for 30 minutes followed by DAB solution for 10 minutes. The slides were then counterstained with hematoxylin and examined under the light microscope. Immunohistochemical evaluation of each protein expression was performed by pathologist. The intensity of immunostaining was evaluated in random 10 fields under 20x magnification. The results were expressed as the percentage of cells with a strong positive staining as follows: ≤10% positive cells − negative (−), between 11% and 50% (+), and ≥51% positive cells (++) [20].

Appropriate positive and negative controls were performed.

## 3. Results

### 3.1. T Cells within Graves' Orbitopathy

In mild type of GO we observed T lymphocytes CD4 positive (T helpers) within the tissue in 6 out of 9 mild cases; mostly T cells were dispersed within the whole tissue and next to the small blood vessels. In 2 specimens T helpers were together with T cytotoxic cells (CD8 positive); however T CD8 were in minority. In 3 mild GO T cells were absent.

#### 3.1.1. T Cells in Severe GO

T lymphocytes were observed in 20 out of 22 severe GO. T cells were mostly CD4 positive T helpers; only in 2 cases we also observed few CD8 positive T cytotoxic cells.

#### 3.1.2. B Lymphocytes in GO

We did not observed presence of B lymphocytes in almost all specimens of both severe and mild GO (with only 1 exception of severe GO, where we observed a focal infiltration of B lymphocytes).

#### 3.1.3. CD68 Expression in Mild GO

In all mild GO tissues we observed presence of CD68 positive cells—both macrophages and fibroblasts. The cells were dispersed in whole tissue, especially in more fibrotic connective tissue, rather than in fat one. In 4 out of 9 cases the cells were numerous and prominent (see Table 2).

#### 3.1.4. CD68 Expression in Severe GO

In all severe GO specimens we observed presence of macrophages and fibroblasts; mostly the cells were numerous and were disseminated in whole tissue (in 15 cases we evaluated the staining as ++). Number of patients and the percentage of CD68 expression within score 0, 1, 2 in examined groups (see Table 3).

#### 3.1.5. COX-2 Expression in Mild and Severe GO

We did not observe COX2 staining, in both mild and severe cases. The explanation of this may by associated with the previous steroid therapy in almost all GO patients.

#### 3.1.6. FGF-*β* Expression in Mild GO

In all mild GO tissues we observed expression of FGF-*β*. The staining was observed mainly in adipocytes and fibroblasts within the tissue. FGF-*β* was also expressed by endothelial cells of the blood vessels within the tissue. However only 2 out of 9 mild GO were evaluated as (++).

#### 3.1.7. FGF-*β* Expression in Severe GO

All of the severe GO cases showed FGF-*β* expression within the adipose tissue as well as in the fibroblasts in the connective tissue. Also FGF-*β* expression was observed in numerous small blood vessels. In almost all of the examined severe GO cases (15 specimens) FGF-*β* expression was estimated as (++). Number of patients and the percentage of FGF-*β* expression within score 0, 1, 2 in examined groups (see Table 3).

#### 3.1.8. TGF-*β* Expression in Mild GO

All of the examined mild GO specimens showed TGF-*β* expression mainly within the fibroblasts of the connective fibrous tissue; however the expression was mostly dispersed.

#### 3.1.9. TGF-*β* Expression in Severe GO

Most of the examined severe GO specimens presented strong and diffused pattern of the TGF-*β* expression, all in the numerous fibroblasts within the connective fibrous tissue.

There was a positive correlation of CAS values with CD4 and FGF-*β*, TGF-*β*, and CD68 expression (see Table 4).

## 4. Discussion

Retrobulbar tissue specimens are generally not available from patients with early, active GO without prior immunomodulatory treatment (with glucocorticoids or radiotherapy).

Avunduk et al. studied 4 biopsy specimens from active GO without prior immunosuppressive treatment [21]. They demonstrated that both CD4+ and CD8+ cells were present, and a significant proportion of them were CD45RO+ cells. Infiltration of OCT (orbital connective tissue) by HLA-Dr+, CD25+, and TNF-*α* cells suggests that Th1-type immune reaction with the interference of proinflammatory cytokine(s) (TNF-*α*) may be important in the pathogenesis of disease [21].

Recently an elevation of CD4 to CD8 ratio and enhanced secretion of IL-6, IL-10, and TNF*α* were detected in PBMCs of GO patients compared with controls [22].

Similar to our study, previously the examined orbital tissue specimens from patients with GO who had received immunosuppressive therapy have shown predominant CD4+ infiltration and only 20–30% of infiltrating cells were CD3+CD8+ cells [23, 24].

Yang et al. examined orbital OCT-derived T cell lines from GO using immunohistochemical methods, and they reported that T cell marker CD3+ could be detected in almost 100% of cases. In addition, T cell lines consisted predominantly of CD4+ cells [24]. Similar results were found by Yang and coworkers who established 104 T cell clones from OCT biopsies of 3 patients with GO and found that approximately 70–80% were CD3+CD4+ and approximately 20–30% were CD3+CD8+ cells [24]. Nevertheless, in both published papers all examined patients had been treated either with systemic steroids and/or orbital radiotherapy. In the previous study, Eckstein et al. have shown the predominant intraorbital CD4+ T cells infiltration in GO with absence of CD8+ and CD20+ B lymphocytes [25]. These results are in agreement with our findings.

Chen et al. found that macrophage infiltration may play an important role in the pathogenesis of GO via overexpression of MCP-1 [26]. The infiltration of macrophages was located primarily around blood vessels and between mature adipocytes. Macrophage infiltration did not attenuate in GO of long duration. They also found that the expression of MCP-1 was higher in GO orbital fat than in the orbital fat of controls [26]. In our study we demonstrated CD68 macrophages' infiltration in fibrous connective and fat tissue more expressed in severe than mild GO (Figures 3(c) and 3(d)). Previously, Eckstein et al. have documented in the orbital tissue an increase of immigrant macrophages CD14 and RFD7 influx [25]. The presence of such cells in GO with strong correlation to disease activity suggests robust proinflammatory secretion on site [26]. Eckstein et al. concluded that these macrophages are recruited from freshly infiltrated monocytes, but not local resident macrophages [25]. Recently it has been found that, in GO, mast cells, monocytes, and macrophages may activate orbital fibroblasts via secretion of especially PDGF-AB and PDGF-BB [27].

**Figure 2:**
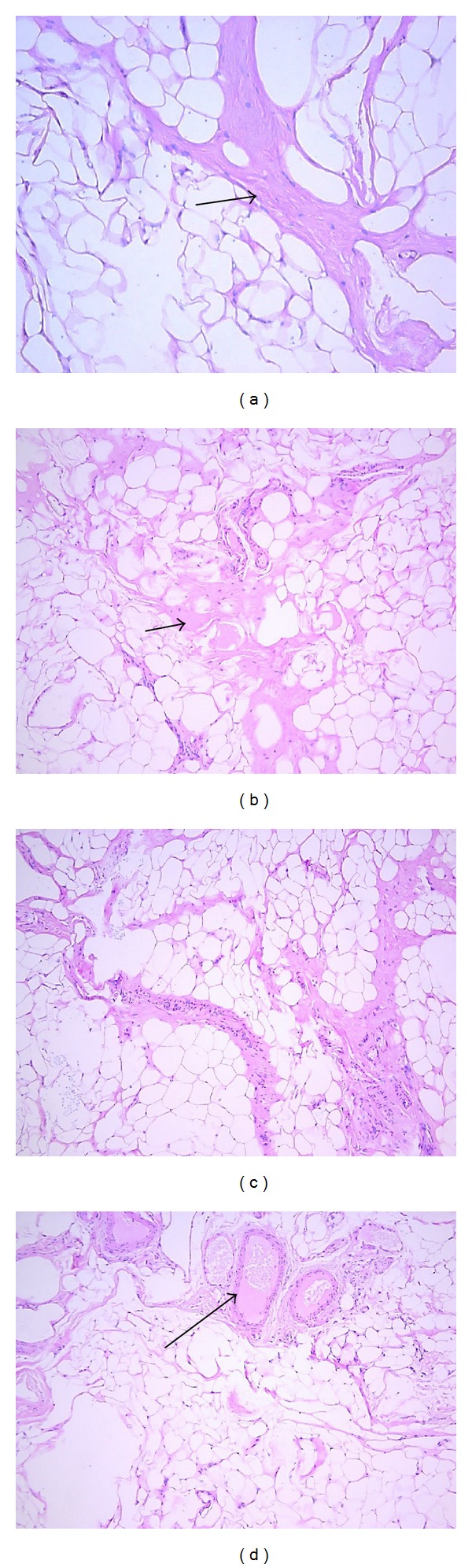
Hematoxylin and Eosin staining in paraffin embedded sections. (a) Mild type of GO. The arrow head shows the area of the intense fibrosis. Magn. 400x, 100x. (b) Mild type of GO. The arrow head shows the small blood vessels. Magn. 400x, 100x. (c) Severe type of GO. Significant proliferation of the fibrous connective tissue within the fat. Magn. 400x. (d) The arrow head shows the prominent blood vessels within the fatty tissue in the severe GO orbitopathy. Magn. 200x.

**Figure 3:**
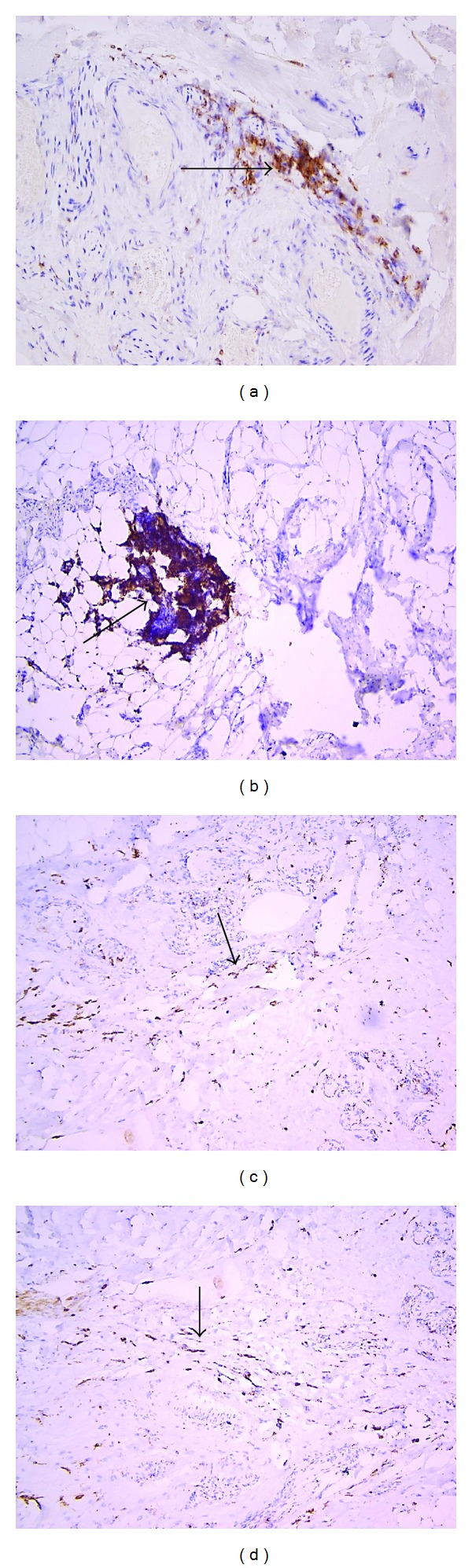
Immunohistochemistry with DAB staining. (a) The arrow head presents the focal cluster of T lymphocyte (CD4+) brown cytoplasmatic staining in the mild type of GO. Magn. 200x. (b) The arrow head shows the cluster of T lymphocytes (CD4+) brown staining in the severe type of GO. Magn. 100x. (c) and (d) The arrow head presents significant fibroblasts and macrophages clusters (CD68+) brown cytoplasmatic staining observed in the fibrous connective area of the adipose tissue of the mild GO (robust 3c) and severe one (d). Magn. 100x.

**Figure 4:**
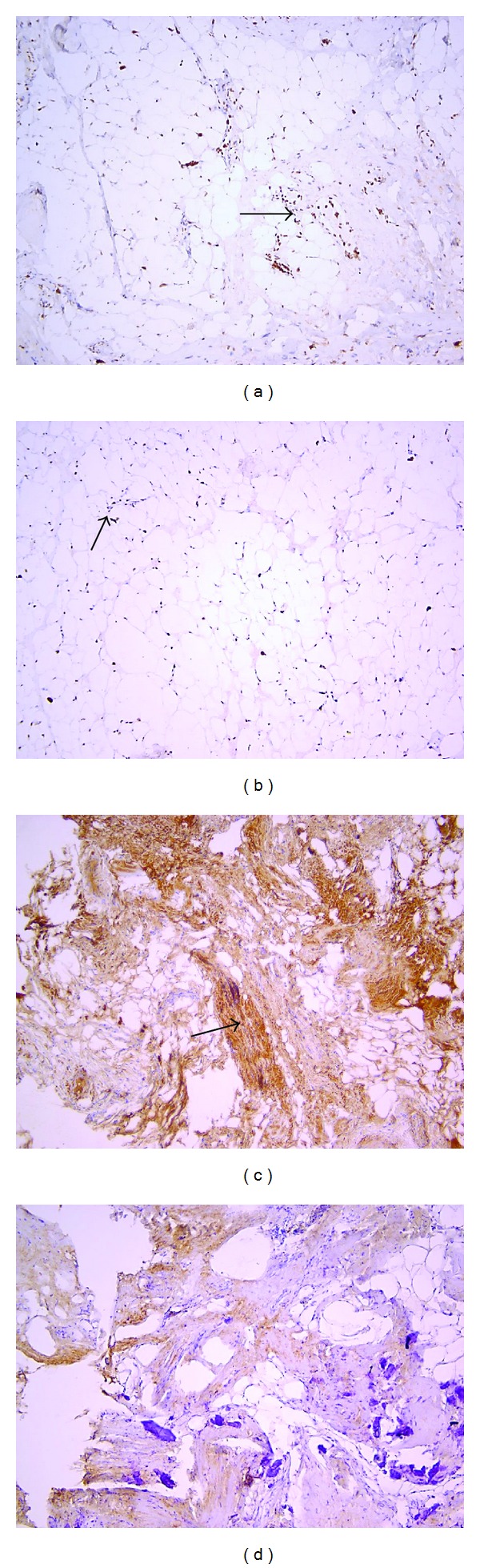
Immunohistochemistry with DAB staining. (a) and (b) FGF-*β* expression observed in the fibroblasts (a) and adipocytes (b) in the connective tissue of the severe GO. Magn. 40x. (c) and (d) A prominent TGF-*β* expression observed in the fibrous connective tissue of the severe (c) and mild (d) GO. Magn. 40x.

Matos et al. have investigated the immunohistochemical expression of growth factors (IGF-1, PDGF-A, PDGF-B, FGF, and VEGF) in patients with Graves' ophthalmopathy [17]. IGF-1 expression was positive in 29.2% of cases. There was a direct relation with higher CAS (clinical activity score) in all of them. When CAS equal or higher than 5 was considered, the percentage of IGF-1 expression was 54.5%. FGF expression was in 5 cases (20.8%) with a direct relation in all those with higher CAS (>5), suggesting its important role in active GO (45.4%). They concluded that in all patients, except one, with positive expression of FGF, IGF-1 and VEGF showed CAS greater than 5, suggesting in this way an important role of these growth factors in the pathogenesis and severity of Graves' ophthalmopathy [17]. Significant proliferation of the fibrous connective tissue in severe GO was seen in our specimens (Figure 2(c)). In addition to our study an increased expression of FGF*β* in the fibroblasts (Figure 4(a)) and adipocytes (Figure 4(b)) was observed in the connective tissue of the severe GO.

Yi and Xu observed high COX2 expression in thyroid-associated ophthalmopathy and Bloise et al. proved treatment of moderate GO with oral sodium diclofenac is a good and safe therapeutic option [28, 29]. Vondrichova et al. showed COX2 overexpression in patients in active phase compared to chronic phase of GO. Moreover, they found that diclofenac, an inhibitor of cyclooxygenases with antagonistic effects on PPAR-gamma, reduced the number of mature adipocytes by approximately 50% [16]. Recently attenuation of interleukin- (IL-) 1*β*-induced cyclooxygenase- (COX-) 2 and prostaglandin (PG)E2 expression in orbital fibroblasts from patients with thyroid-associated ophthalmopathy (TAO) has been proven to decrease inflammation [30]. This statement would be in agreement with our results, showing no COX-2 expression in our specimens, since most of our studied patients received prior to orbital decompression glucocorticosteroids that may had hampered the inflammation mandated by COX2.

## 5. Conclusions

Macrophages and CD4 T lymphocytes are both engaged in the active/severe and long stage of inflammation in the orbital tissue. FGF-*β* and TGF-*β* expression may contribute to tissue remodeling, fibrosis, and perpetuation of inflammation in the orbital tissue of GO especially in severe GO. COX2 pathway may be hampered by systemic steroids treatment.

## Figures and Tables

**Table 1:** Antibodies used in the study for immunohistochemical staining.

Antibody	Type of antibody	Dilution
CD4	Monoclonal mouse anti-human CD4 DAKO cytomation clone 4B12	1 : 40
CD8	Monoclonal mouse anti-human CD8 DAKO cytomation clone C8/144B	1 : 50
CD20	Monoclonal mouse anti-human CD20cy DAKO cytomation clone L26	1 : 200
CD68	Monoclonal mouse anti-human CD68 DAKO cytomation clone PG-M1	1 : 100
TGF*β*	Rabbit polyclonal TGF*β*1 (V) antibody: sc-146 Santa Cruz Biotechnology	1 : 50
FGF*β*	Rabbit polyclonal FGF-1 antibody (H-125): sc-7910 Santa Cruz Biotechnology	1 : 100
COX2	Monoclonal mouse anti-human COX-2 DAKO cytomation clone CX-294	1 : 50

**(a):** 

Number	Classification	CD4/CD8	CD20	CD68	COX-2	FGF	TGF
1	Contrl.	0-0	0	1	0	0	0
2	Contrl.	0-0	0	0	0	0	0
3	Contrl.	0-0	0	0	0	0	0
4	Contrl.	0-0	0	0	0	0	0
5	Contrl	0-0	0	0	0	0	0
6	Contrl	0-0	0	1	0	1	0
7	Contrl.	0-1	1	1	0	1	0

**(b):** 

Number	Classification	CAS score	CD4/CD8	CD20	CD68	COX-2	FGF	TGF
1	Mild GO	1	0-0	0	1	0	1	1
2	Mild GO	3	1-0	0	2	0	2	1
3	Mild GO	2	1-0	0	1	0	1	1
4	Mild GO	3	1-0	0	2	0	2	1
5	Mild GO	1	1-0	0	1	0	1	1
6	Mild GO	3	1-1	0	2	0	1	1
7	Mild GO	1	1-1	0	1	0	1	1
8	Mild GO	3	0-0	0	2	0	1	2

**(c):** 

Number	Classification	CAS score	CD4/CD8	CD20	CD68	COX-2	FGF	TGF
1	Severe GO	10	1-0	0	2	0	2	2
2	Severe GO	10	1-0	0	2	0	2	2
3	Severe GO	9	1-0	0	2	0	2	1
4	Severe GO	8	1-0	0	2	0	2	2
5	Severe GO	7	1-0	0	1	0	1	1
6	Severe GO	10	1-0	0	2	0	2	2
7	Severe GO	10	1-0	0	2	0	2	2
8	Severe GO	9	1-0	2	2	0	2	2
9	Severe GO	10	1-1	0	2	0	2	2
10	Severe GO	10	2-1	0	2	0	2	2
11	Severe GO	5	1-0	0	1	0	1	1
12	Severe GO	10	1-0	0	2	0	2	2
13	Severe GO	10	1-0	0	2	0	2	2
14	Severe GO	6	1-0	0	2	0	1	1
15	Severe GO	5	0-0	0	1	0	1	1
16	Severe GO	5	0-0	0	1	0	1	1
17	Severe GO	5	1-0	0	1	0	1	1
18	Severe GO	8	1-0	0	1	0	2	1
19	Severe GO	10	1-0	0	2	0	2	2
20	Severe GO	7	1-0	0	2	0	1	2
21	Severe GO	5	1-0	0	1	0	2	2
22	Severe GO	10	1-0	0	2	0	2	2

Legend for Tables 2(a), 2(b), and 2(c): immunohistochemistry score:

0-less than 10% positive in 10 representative high power fields (HPF).

1-10%–50% positive cells in 10 HPF.

2-more than 50% positive cells in 10 HPF.

Immunohistochemistry was done using DAB chromogen (brown staining). Ctrl: controls.

**(a):** 

FGF expression	Control	Mild GO	Severe GO	Sum
≤10% no.	5	0	0	5
[%]	71.43%	0.00%	0.00%	
(10%; 50%) no.	2	6	7	15
[%]	28.57%	75.00%	31.81%	
>50% no.	0	2	15	17
[%]	0.00%	25.00%	68.18%	

**(b):** 

CD68 expression	Control	Mild GO	Severe GO	Sum
≤10% no.	4	0	0	4
[%]	57.14%	0.00%	0.00%	
(10%; 50%) no.	3	4	7	14
[%]	42.86%	50.00%	31.81%	
>50% no.	0	4	15	19
[%]	0.00%	50.00%	68.18%	

**Table 4:** Pearson's correlation of CAS values with examined parameters.

	CAS	
	*R* value	*P* value
CD4 expression	.4071	*P* = .028
CD8 expression	−.0632	*P* = .745
CD20 expression	.1432	*P* = .459
CD68 expression	.6017	*P* = .001
FGF expression	.7311	*P* = .0001
TGF expression	.7213	*P* = .0001

## Abbreviations

CD: Cluster of differentiation
CAS: Clinical activity score
COX2: Cyclooxygenase 2
FGF-*β*: Fibroblast growth factor
GO: Graves' orbitopathy
MCP-1: Monocyte chemoattractant protein
RANTES: Regulated on activation normal T cell expressed and secreted
TGF-*β*: Transforming growth factor-*β*.
